# Medical and Physical Intelligence

**Published:** 1823-09

**Authors:** 


### MEDICAL AND PHYSICAL INTELLIGENCE.

### MORBID ANATOMY.

1. *Umbilical Tumor.*—Dr. Duchateau was called to examine a new-born male child, in consequence of the alarm of the midwife at perceiving a large tumor in the umbilical region. It was regularly spherical in shape, and so transparent, that the parts it contained were distinctly seen through its parietes: part of the liver, ileum, and mesentery, were thus distinguished; the pulsation of some of the arteries, and the peristaltic motion of the intestines, being likewise perceptible. The tumor measured four inches from above downwards, and three and a half inches in breadth. Care was taken to prevent any injury from friction or compression, and the infant lived eight days.

This case bears considerable analogy to one communicated by Breschet to the Academy of Medicine; at the same time it differs in this, that in the latter the tumor was formed by the lesion and separation of the abdominal parietes, while in Duchateau's case it appeared to be formed by the extreme dilatation of the navel-string, just before it arrived at the umbilicus. This phenomenon occurred so long ago as 1784, at the time when the celebrated Pilate des Roziers was attracting the wonder of France with his balloons, and, as the mother had gone to Versailles, at an early period of pregnancy, to witness one of his ascents, a ready explanation of this monstrosity was afforded; and received such general credit among the gossips of the day, that the Baron de Breteuil, then prime minister, was obliged to interfere to prevent the propagation of human balloons.—*Bulletin de la Societé Medicale d'Emulation,* Juin.

---

THERAPEUTICS.

2. *New Treatment of Croup.*—Professor RECAMIER has informed the Academy of Medicine at Paris, that he has lately been successful in treating cases of croup, with threatened suffocation, by means of milk and water injected by the mouth and nose at the same time, so as to excite violent convulsions of the throat and muscles of the larynx. This plan has been tried in three cases, in all of which portions of false membrane were expelled. It appears, however, that one of the little patients died; and, although this is accounted for by a "dissolution" of the stomach, we are not quite convinced that the injections may not have had something to do with it. *Anceps quam nullum* is the only principle on which so precarious a remedy can be justified.—(*Revue Medicale,* Juin.)

---

PATHOLOGY.

3. *Rupture of the Internal Coats of an Artery.*—Mr. LIZARS relates the case of a gentleman who was travelling on the mail-coach, which was overturned, and his leg got beneath the iron railing. The coach was dragged for a short distance while his limb was in this position, so that it was severely lacerated. On examination, it was discovered that he had been bleeding freely, and that his leg was dreadfully shattered; the ham was completely exposed to the bone, exhibiting the living nerves, blood-vessels, and muscles, as if they had been displayed for demonstration. The popliteal artery felt like a cord, and the leg below was cold and lifeless. The skin was ruffled down to the ankle-joint, like a stocking. Amputation was performed, and the patient ultimately did well.

"Previous to examining the amputated limb, I attempted to inject the popliteal artery from the upper or proximal orifice, but little or no injection entered. I then traced the artery, and found the articular branches and those to the heads of the gastrocnemius externus muscle with open mouths, except one which had a clot of blood in it. On arriving at the division of the popliteal artery, the anterior tibial, the posterior tibial, and fibular branches, were empty and collapsed. The trunk of the popliteal, immediately above its division into posterior tibial and fibular arteries, was then laid open upwards, when the serous and muscular coats were found ruptured across, a portion of them insulated and plugging up the vessel, a clot of blood proximal in the artery, and blood effused in the cellular coat, which had remained comparatively sound and entire. The integuments were detached downwards to the ankle-joint; the muscles, both on the anterior or patellar, and on the posterior or popliteal aspects of the leg, were distended with effused blood; several parts of the cellular substance were also injected with blood; and a considerable quantity of blood was found in the knee-joint. The ham was exposed to the depth of the popliteal ligament, or downwards to the heads of the gastrocnemius internus muscle. The posterior tibial and peroneal nerves were entirely exposed; so were the popliteal vein and artery, which were loosely insulated. Several muscular veins, together with the saphena minor, were lacerated; and the heads of the gastrocnemius externus were extensively injured.

"The effect of the contusion of this artery corroborates what had been advanced by Dessault, Morand, and Petit. We have here, first, the rupture of the two internal coats, as proved by Dessault, Bichat, Drs. Thomson and Jones; secondly, the *crispation* of Morand; and, thirdly the clot of blood both internally and externally, or the *couvercle* and *bouchon* of Petit; all contributing to stem the hemorrhage, and prevent instant death. The muscular branch, which had the clot of blood in it, also shows the method which nature adopts to suppress bleeding. There was no coagulated lymph, as the vasa vasorum and vessels of the sheath of the artery were destroyed."—(*Edinburgh Med. and Surg. Journal*, July.)

### SURGERY.

4. *Extirpation of Part of the Lower Jaw.*—M. DUPUYTREN exhibited, at the Academy of Medicine, in May, a girl of twelve years old, in whom he had removed the front of the lower jaw for an osteo-sarcoma. Although the extirpation extended from the first of the molares on one side to the canine tooth of the other, yet the lip and chin presented a pretty regular cicatrix, and her countenance was much more agreeable than before the operation, which was not attended with any accident.—(*Revue Medicale*, Juin.)

5. *Urinary Calculus, weighing ten Ounces, expelled by a natural Process.*—M. GÉRARD, of Beauvais, has lately published a case under this title. A man, sixty years of age, and of good constitution, had been attacked, when about five-and-twenty, with a feeling of heat in the urinary passages, and an increased diuresis, which was unaccompanied by pain. These circumstances continued for six years, and then gave place to dysuria, with acute pain and irregular fever: the urine at this time flowed *guttatim,* and was mixed with blood and pus. The patient remained for nearly twenty-two years subject to these attacks, but following his usual occupations during the intervals, till 1810, when retention of urine came on, with violent fever and delirium. The surgeon, who was now called, attempted in vain to introduce a sound, while the efforts to evacuate the contents of the bladder became so violent as to produce an inguinal hernia. An abscess formed in the perineum, which was opened on the left side of the raphe, and a great quantity of pus evacuated. As the urine now flowed by this opening, fresh attempts were made to introduce a sound into the bladder, but without success, an invincible resistance being experienced at the neck. Several smaller abscesses subsequently formed, and gave rise to urinary fistulæ. After about eight months' suffering, the patient recovered so far as to resume his usual occupations, although walking was rendered somewhat difficult by a tumor, which, lodging partly in the left groin (*aine*), impeded the movement of the thigh. In July, 1822, an enormous abscess was formed, which involved the scrotum and penis: this was treated with emollient applications, and suppuration was established. In consequence, however, of the violence of the inflammation, or perhaps from the urine coming in contact with the parts, some gangrenous points formed, and, when these fell off, (July 26th,) a large round body was evacuated, by an effort compared by the patient to labour. This body was pear-shaped, with a granulated surface, and weighed ten ounces: at present it weighs only seven and a half. Its circumference is seven and a quarter inches at the middle; six and a half at the larger end, and four and a half at the smaller; it is three and a half inches in length. The patient gradually recovered, suffering no inconvenience except from a fistulous opening.

MM. GAULTIER DE CLAUBRY and DESRUELLES, who report upon this case, are of opinion that the calculus was extra-vesical, and probably was situated in the scrotum or perineum.—(*Bulletins de la Societé Medicale d'Emulation de Paris,* Mai 1823.)

6. *Method of arresting Mercurial Salivation.*—M. SOMMÉ, principal surgeon to the Civil Hospital at Antwerp, announces that this consists in the use of a gargle made with acetate of lead dissolved in water, (*une once d'acetate de plomb liquide dans deux livres d'eau*). It is, according to his observations, very efficacious, being much preferable to alum or borax gargles, or sulphur taken internally. If, says he, M. CULLERIER speaks of it with indifference in the Dictionnaire des Sciences Médicales, it is because the trials made at the Venereal Hospital in Paris could not but fail, from the weakness of the solution. This remedy has the disadvantage of blackening the teeth, but is said to heal quickly those ulcerations of the mouth which generally resist other means. In the ulcers of the tonsils and palate, which sometimes follow mercurial courses, M. Sommé touches the parts with a hair-pencil, charged with the pure liquid of acetate of lead.—(*Archiv. gener. de Medecine,* Avril.)

7. *Excision of Encysted Tumors.*—Mr. M'GHIE suggests an improvement in the excision of those species of encysted tumors which have been denominated the Melicerous and Atheromatous.

"It is well known that the extirpation of the former, especially, of these kinds of swellings, is often rendered extremely tedious and difficult from the tenuity of the cyst, and the fluidity of its contents. Notwithstanding the utmost caution and adroitness on the part of the surgeon, the sac will at times be opened in dissecting it from the contiguous parts; and, by its collapsing, the completing of the operation is much embarrassed, if not altogether prevented.

"To obviate so unpleasant an occurrence, and also to enable the operator to remove the whole of the disease with the greater certainty and expedition, I would advise, whenever there is the least reason to suppose that the foregoing difficulties will be encountered, that the sac should be opened with an abscess lancet; its contents pressed out; and, to clear it completely of these, some tepid water is to be thrown into it with a large syringe. A thin mixture of sulphate of lime (plaster of Paris) and water is then to be injected into the cyst, until it is distended to its former bulk; and, as this injection hardens immediately, it will occasion no delay in the operation: on the contrary, from the contents of the tumor being now a calcareous concrete, the detachment of its cyst will be found a very easy matter. The amount of pain, therefore, which would have been suffered in the removing of it, will be lessened; and, what is of still greater importance, there can be no risk of leaving any portion of it behind, to become the germ of a troublesome, and perhaps very serious, disease.

"The syringe which I would recommend to be used ought to be capable of containing half a pint, and have a long conical mouthpiece, with which the operator is to be careful that he fills the aperture into the cyst, whilst he is throwing in the mixture of the sulphate of lime, so that none of the injection shall escape into the cellular substance external to the sac; and he is not to withdraw the syringe until this hardens, which it will do in the course of a few seconds. It is almost unnecessary to add, that the injection should not be prepared until it is just going to be used; for it becomes solid in a very short time, from the strong affinity which the sulphate of lime has for water."—(*Edinburgh Med. and Surg. Journal,* July.)

8. *Successful Case of Inguinal Aneurism.*—The operation of tying the external iliac artery for the cure of inguinal aneurism, was performed by Dr. J. C. WARREN, in the Massachusetts General Hospital, on the 20th February last, with some interesting circumstances. The aneurismal tumor was large, and extended upward into the cavity of the abdomen. When the muscles had been divided, and the abdominal cavity opened, the aneurism was seen rising so high as to conceal the iliac artery. From the anterior and inner part of the sac arose the epigastric, giving origin to the obturator artery, both of them greatly enlarged; the epigastric trunk being equal to the common size of the brachial. From the outer part of the sac rose the circumflexa ilii, also considerably enlarged. The epigastric interfered much with the necessary dissection of the great artery; and the iliac was exposed higher than usual, but the ligature was passed round it without difficulty. The event of this operation was favourable. The limb never lost its warmth, sensation, nor motion. The patient was restrained to his bed with difficulty till the ligature had separated: on the nineteenth day he was allowed to rise; on the following days walked about, and afterwards recruited rapidly.—(*New-England Journal.*)

---

### CHEMISTRY.

9. *Galvanic Apparatus.*—A new and powerful apparatus has been constructed at the London Institution, by the ingenious W. H. PEPYS, Esq. It consists of a single sheet of copper and one of zinc, each fifty feet long and two broad. They are wound round a wooden centre, and kept apart by pieces of interposed hair-lines. The coil and its counterpoise are suspended by a rope over a tub of dilute acid. When lowered into the tub, its electricity is so low as not to affect the electrometer; even a bit of charcoal serves to insulate it; and it can hardly ignite an inch of platinum wire, $1.31^2$ of an inch in diameter: but, when the poles are connected by a copper wire, $1.8^2$ of an inch in diameter, and eight inches long, it becomes hot, is most powerfully magnetic, and admirably adapted for all electro-magnetic experiments.—(*Philosophical Mag.* June.)

10. *Abstract of Experiments on the Fusion of Plumbago, Anthracite, and the Diamond.*—Professor SILLIMAN has succeeded in fusing and volatilizing plumbago, anthracite, and the diamond, by means of Dr. HARE's galvanic deflagrator.

"From a piece (says he,) of very fine plumbago, from Carolina, I sawed small parallelopipeds, about one-eighth of an inch in diameter, and from three fourths of an inch to one inch and a quarter in length: these were sharpened at one end, and one of them was employed to point one pole of the deflagrator, while the other was terminated by prepared charcoal. The best were obtained when the plumbago was connected with the copper, and prepared charcoal with the zinc pole. The spark was vivid, and globules of melted plumbago could be discerned, even in the midst of the ignition, forming and formed upon the edges of the focus of heat. In this region, also, there was a bright scintillation, evidently owing to combustion, which went on where air had free access, but was prevented by the vapour of carbon, which occupied the highly luminous region of the focus between the poles, and of the direct route between them. Just on and beyond the confines of the ignited portion of the plumbago, there was formed a belt of a reddish-brown colour, a quarter of an inch or more in diameter, which appeared to be owing to the iron, remaining from the combustion of the carbon of that part of the piece, and which, being now oxidized to a maximum, assumed the usual colour of the peroxide of that metal.

"In various trials, the globules were formed very abundantly on the edge of the focus, and in several instances were studded around so thickly as to resemble a string of beads, of which the largest were of the size of the smallest shot; others were merely visible to the naked eye, and others still were microscopic. No globule ever appeared on the point of the plumbago which had been in the focus of heat; but this point presented a hemispherical excavation, and the plumbago there had the appearance of black scoriæ, or volcanic cinders. These were the general appearances at the copper pole occupied by the plumbago.

"On the zinc pole occupied by the prepared charcoal, there were very peculiar results. The pole was in every instance elongated towards the copper pole, and the black matter accumulated there presented every appearance of fusion, not into globules, but into a fibrous and striated form, like the half-flowing slag found on the upper currents of lava. It was evidently transferred, in the state of vapour, from the plumbago of the other pole, and had been formed by the carbon taken from the hemispherical cavity. It was so different from the melted charcoal, described in my former communications, that its origin from the plumbago could admit of no reasonable doubt. I am now to state other appearances, which have excited in my mind a very deep interest. On the end of the prepared charcoal, and occupying frequently an area of a quarter of an inch or more in diameter, were found numerous globules of perfectly melted matter, entirely spherical in their form, having a high vitreous lustre, and a great degree of beauty. Some of them, and generally they were those most remote from the focus, were of a jet black, like the most perfect obsidian; others were brown, yellow, and topaz-coloured; others still were greyish-white, like pearl-stones, with the translucence and lustre of porcelain; and others still limpid like flint-glass, or, in some cases, like hyalite or precious opal, but without the iridescence of the latter. Few of the globules upon the zinc pole were perfectly black, while very few of those on the copper pole were otherwise. In one instance, when I used some of the very pure English plumbago, (believed to be from Borrowdale,) white and transparent globules were formed on the copper side.

"I detached some of the globules, and, partly bedding them in a handle of wood, tried their hardness and firmness: they bore strong pressure without breaking, and easily scratched, not only flint-glass, but window-glass, and even the hard green variety, which forms the aquafortis bottles.

"My first trials were made by placing small *diamonds* in a cavity in charcoal; but the support was, in every instance, so rapidly consumed, that the diamonds were speedily displaced by the current of gas. I next made a chink in a piece of solid quick-lime, and crowded the diamond into it. This proved a very good support; but the effulgence of light was so dazzling, that although, through green glasses, I could steadily inspect the focus, it was impossible to distinguish the diamond in the perfect solar brightness. This mode of conducting the experiment proved, however, perfectly manageable; and a large dish, placed beneath, secured the diamonds from being lost, (an accident which I had more than once met with,) when suddenly displaced by the current of gas. As, however, the support was not combustible, it remained permanent, except that it was melted in the whole region of the flame, and covered with a perfect white enamel of vitreous lime. The experiments were frequently suspended, to examine the effect on the diamonds.

They were found to be rapidly consumed, wasting so fast that it was necessary, in order to examine them, to remove them from the heat at very short intervals: they exhibited, however, marks of incipient fusion. My experiments were performed upon small wrought diamonds, on which there were numerous polished facets, presenting extremely sharp and well-defined solid edges and angles. These edges and angles were always rounded, and generally obliterated. The whole surface of the diamond lost its continuity, and its lustre was much impaired; it exhibited innumerable very minute indentations, and intermediate and corresponding salient points; the whole presenting the appearance of having been superficially softened and indented by the current of gas, or perhaps of having had its surface unequally removed by the combustion. In various places, near the edges, the diamond was consumed, with deep indentations. These results seem to indicate that, were the diamond a good conductor, it would be melted by the deflagrator; and, were it incombustible, a globule would be obtained by the compound blowpipe."—(*Edinburgh Philosophical Journal.*)

---

### PHARMACY.

11. *Difference between Indigenous and Levant Opium.*—M. Ricard Duprat, a chemist at Toulouse, informs us that a lady of that city, who suffered excruciating torment from a cancer, was unable to procure relief from opium as usually given, although she took as much as an ounce in the week. Extract of poppy-heads was substituted, with the best effects, producing freedom from suffering, and occasionally tranquil sleep. The extract was prepared as follows:—The poppy-heads were carefully cleaned of the seeds and stalks, bruised in a mortar, and infused in cold water for twelve hours; they were then repeatedly boiled in fresh quantities of water; the liquor was passed through linen of very close texture, and evaporated. Of the extract thus obtained, an ounce was divided into forty-eight pills; but we are not informed how many of these the lady took for a dose. M. Duprat, in reasoning upon this subject, gives it as his opinion that it is nearly impossible to free the opium of commerce from a certain quantity of resin, while the native extract contains scarcely any. If then, says he, it be true that the resins in general, and that of opium in particular, act by producing irritation, can we be astonished at obtaining more satisfactory results from the one than the other?

M. Robiquet, in remarking upon this communication, gives a more scientific account of the matter. Vauquelin has ascertained that the extract of poppies reared in these parts contains *morphine*, although in smaller quantity than the opium of the Levant; and many practitioners of respectability have described the indigenous opium as purely sedative in its effects. The difference in the operation of the two varieties of this drug arises, according to Robiquet, from the circumstance of the *narcotin* being present in the Levant opium, and not in that produced in this part of Europe; and, so far as we know at present, the calming effects depend upon the salts of morphine.—(*Bulletins de la Societé Medicale d'Emulation*, Mai 1823.)

MISCELLANEOUS.

12. *Fumigating Baths.*—We have lately had the pleasure of inspecting the fumigating baths recently established by Mr. Green, in Bury-street, St. James's. They are in many respects greatly superior to those hitherto established in this country, as well as to those originally constructed in Paris; especially with reference to the situation of the fire, which is here upwards of five feet below the baths, whereas the usual distance does not much exceed one foot. In consequence of this improvement, the bath is heated much more gradually, and the patient is enabled to remain in it for a longer period. We saw a severe case of Impetigo inveterata, now under Mr. Green's care, in which the beneficial effects of the sulphureous vapour were strikingly displayed.

13. *Death of a Child, eleven Months old, supposed to have been produced by the Milk of its Mother.*—In April, 1821, a person at Munster quarelled with a soldier, who lodged in his house: the latter drew his sabre, and attacked his host, whose wife, terrified at the danger of her husband, rushed upon his adversary, wrested the weapon from his hand, and threw it to a distance. By this time others had arrived, and the combatants were separated. The woman then, being still greatly agitated by the occurrence, took her infant, who was quite well, out of its cradle, and applied it to her breast. The child quitted the nipple with marks of inquietude, sighed, and remained lifeless in the mother's arms! Dr. Tourtual, of Munster, was called within a quarter of an hour, and administered all the assistance in his power, but without avail. The influence of the mother's milk upon the child has long been known: the passions of nurses, in particular, are unfavourable to their infants, who are almost always affected, under such circumstances, with restlessness, colic, diarrhœa, vomiting, &c.; but we are not aware of any case similar to the present, (related by M. Tourtual,) in which the milk would seem to have acted as a quick and powerful poison. It is to be regretted that no account is given of any examination after death.—*(Journal der Practischen Heilkunde, von* Hufeland, 1823.)

13. *Sulphur as a Preservative against Measles.*—During the winter of 1817, the measles prevailed epidemically at Munster, at which time M. Tourtual had occasion to remark that the children affected with itch, and who were using sulphur externally and internally, were exempt from the epidemic. He attributed this circumstance to the presence of another cutaneous disease, and gave it all the merit of the prophylactic virtue. In 1822, a fresh epidemic of measles occurred again, and the disease was preceded for many days by a convulsive cough. For this symptom M. Tourtual prescribed a mixture of flowers of sulphur and white sugar, of which the children took half a tea-spoonful,—more or less according to their age. Many trials were made on children of different families and different ages, and *all* who took the remedy in time escaped the disease.—*(Ibid.)*

### METEOROLOGICAL JOURNAL,

*From July 20, to August 19, 1823.*

By Messrs. William Harris and Co. 50, Holborn, London.

| | MOON. | Rain gauge | THERM. | | | BAROM. | | DE LUC'S HYG. | | WIND. | | ATMOSPHERIC VARIATION. | | |
|---|---|---|---|---|---|---|---|---|---|---|---|---|---|---|
| | | | 9 A.M. | MAX. | MIN. | 9 A.M. | 10P.M. | 9 A.M. | 10P.M. | 9 A.M. | 10P.M. | 9 A.M. | 2 P.M. | 10P.M. |
| July | | | | | | | | | | | | | | |
| 20 | | .15 | 69 | 75 | 65 | 29.80 | 29.74 | 72 | 70 | SSW | S | Cloud. | Fine | Fine |
| 21 | | | 66 | 69 | 62 | 29.50 | 29.44 | 80 | 75 | SSE | WSW | Cloud. | Rain | Cloud. |
| 22 | | .05 | 61 | 67 | 55 | 29.63 | 29.70 | 74 | 74 | WSW | WSW | Fine | Sho'ry | Fine |
| 23 | ○ | .03 | 60 | 65 | 53 | 29.50 | 29.23 | 82 | 85 | S | SW | Rain | Rain | Rain |
| 24 | | .22 | 58 | 67 | 53 | 29.50 | 29.65 | 75 | 80 | W | WSW | Cloud. | Fine | Fine |
| 25 | | | 57 | 65 | 56 | 29.64 | 29.50 | 71 | 85 | SW | SSE | Cloud. | Fine | Rain |
| 26 | | | 55 | 65 | 54 | 29.43 | 29.54 | 89 | 74 | W | NW | Rain | Sho'ry | Cloud. |
| 27 | | .40 | 55 | 66 | 53 | 29.60 | 29.71 | 70 | 75 | W | W | Cloud. | Fine | |
| 28 | | | 55 | 67 | 54 | 29.68 | 29.65 | 85 | 80 | W | S | Rain | Fair | Fair |
| 29 | | .12 | 59 | 64 | 54 | 29.63 | 29.58 | 87 | 83 | SW | SW | | Sho'ry | |
| 30 | ☾ | | 60 | 67 | 57 | 29.60 | 29.63 | 79 | 80 | SW | WSW | Fine | Fine | Rain |
| 31 | | .03 | 60 | 69 | 55 | 29.70 | 29.85 | 75 | 76 | SW | WSW | | | Fine |
| Aug | | | | | | | | | | | | | | |
| 1 | | | 59 | 68 | 60 | 29.90 | 29.77 | 77 | 80 | SSW | SSW | Cloud. | | Cloud. |
| 2 | | | 59 | 70 | 58 | 29.81 | 29.80 | 75 | 77 | S | SW | Rain | | Fine |
| 3 | | .03 | 62 | 67 | 59 | 29.65 | 29.55 | 87 | 80 | S | SSW | Overc. | Rain | Rain |
| 4 | | .30 | 64 | 69 | 68 | 29.50 | 29.62 | 76 | 77 | SSW | SW | Fine | Fine | Fine |
| 5 | | | 65 | 67 | 65 | 29.62 | 29.63 | 72 | 75 | SW | SW | | Sho'ry | |
| 6 | ● | .02 | 64 | 66 | 63 | 29.60 | 29.60 | 72 | 71 | SW | SSW | | | |
| 7 | | | 60 | 64 | 57 | 29.70 | 29.64 | 72 | 74 | SW | S | | | |
| 8 | | | 58 | 64 | 51 | 29.60 | 29.62 | 82 | 73 | SW | W | Rain | | |
| 9 | | .15 | 55 | 61 | 48 | 29.72 | 29.86 | 75 | 76 | WSW | W | Fine | | |
| 10 | | .10 | 56 | 63 | 49 | 29.93 | 29.86 | 89 | 100 | SSW | SSW | Rain | Rain | Rain |
| 11 | | .11 | 60 | 65 | 58 | 29.83 | 29.80 | 95 | 94 | SW | SSW | Cloud. | Cloud. | Cloud. |
| 12 | | | 60 | 75 | 62 | 29.73 | 29.66 | 73 | 75 | SSW | S | Fine | Fine | Fine |
| 13 | ☽ | | 65 | 76 | 60 | 29.50 | 29.54 | 72 | 75 | SW | SSW | | | |
| 14 | | | 64 | 74 | 57 | 29.63 | 29.65 | 74 | 73 | WSW | SW | | | Li. Ra. |
| 15 | | .04 | 60 | 74 | 49 | 29.64 | 29.50 | 72 | 76 | SSW | WSW | | | |
| 16 | | | 57 | 65 | 49 | 29.37 | 29.49 | 80 | 77 | S | SW | Sho'ry | Sho'ry | |
| 17 | | .22 | 60 | 63 | 48 | 29.64 | 29.74 | 65 | 76 | WSW | S | Fine | Cloud. | Cloud. |
| 18 | | | 58 | 64 | 49 | 29.71 | 29.66 | 75 | 95 | ESE | S | Cloud. | | Rain |
| 19 | | | 64 | | | 29.62 | 29.64 | 92 | 94 | SSW | SSW | | Rain | |

The quantity of rain during the month of July, was 2 inches and 37.100ths.

